# The compensation incentive effect of athletes: A structural equation model

**DOI:** 10.3389/fpsyg.2022.1034855

**Published:** 2022-11-18

**Authors:** Huan Zhao, Zhaoxia Liu, Susu Zhang, Feiyan Xiao, Meng Liu, Ruiyuan Li, Liqing Zhang, Chengcheng Xu

**Affiliations:** ^1^Sports Coaching College, Beijing Sport University, Beijing, China; ^2^Graduate School, Hunan University, Gwangju Metropolitan City, South Korea; ^3^Sports Department, Chengyang Experimental Primary School, Shandong, China; ^4^Faculty of Physical Education, Tomsk State University, Tomsk, Russia; ^5^Sports Department, The Branch of the High School Afflicted to Renmin University of China, Beijing, China

**Keywords:** athlete, salary structures, compensation satisfaction, compensation incentive effect, structural equation model

## Abstract

This study explores the compensation incentive effect of athletes. Based on the related literature, we proposed theoretical hypotheses on the compensation incentive effect and established an assessment index system of the compensation incentive effect for athletes. A structural equation model was used to test the survey data of 352 athletes in six provinces to discover the truth of the compensation incentive effect. The results suggested that direct economic compensation satisfaction, direct non-economic compensation satisfaction, and indirect non-economic compensation satisfaction had significant positive effects on the compensation incentive effect of athletes, while indirect economic compensation satisfaction showed no significant effect. Moreover, the evaluation results of athletes’ compensation incentive effect showed that direct economic compensation satisfaction contributed the most to the influence factor of the compensation incentive effect. Therefore, the evaluation of athletes’ compensation incentive effect should focus on variables of direct economic compensation satisfaction, i.e., basic compensation satisfaction, bonus income satisfaction, and subsidy satisfaction. Finally, some strategies and recommendations were suggested to improve the compensation design for athletes.

## Introduction

China is transforming from a large sports country to a strong sports country, as evidenced by its glorious developing course from the breakthrough of zero gold medals at the 1984 Los Angeles Olympic Games in the United States to the leap to the top of medal ranking at the 2008 Beijing Olympic Games and then to the success in the 2022 Beijing Winter Olympic Games. A key to improving the level of sports in China, a competitive country in the field of sports, is to solve the income distribution problem for athletes, especially in terms of salary incentives. The evolution of the athletes’ remuneration system in China can be divided into two stages: during the planned economy, the state implemented the athletes’ remuneration system with the standard salary as the income source under the influence of “egalitarianism”; during the market economy when the state gradually implemented the salary distribution method based on the distribution of labor, athletes’ remuneration system was improved with the deepening reform of the sports system.

Although athletes’ compensation mechanism in China tends to be rationalized, with improved salary structures and increased compensation levels, there are still some shortcomings in the overall design of the compensation system. The most obvious deficiencies are reflected in the excessive income gap among athletes (Zou and Cui, 2014), unreasonable bonus distribution, and inadequate welfare systems such as social security and commercial insurance. Therefore, how to optimize the salary structure of athletes, improve the salary distribution system and motivate them to train has become the core problem that needs to be solved. Based on the literature review and field research, this paper introduces the structural equation modeling method to evaluate athletes’ compensation incentive effect, thus providing theoretical reference and a practical basis for scientifically and reasonably reconstructing athletes’ salary structure in China.

## Literature review

Compensation is considered to be the sum of various monetary incomes and benefits that employees receive from their employers (Milkovich and Newman, 2004). With the diversified development of compensation forms, compensation includes not only the material rewards paid to employees by the organization but also non-material rewards such as development and promotion opportunities, social status, and career achievements (Steinberger, 2018; Tan et al., 2019). The compensation structure has changed from single “monetary compensation” to compound “comprehensive compensation.” Comprehensive compensation usually refers to economic compensation, including direct and indirect economic compensation (Gulyani and Sharma, 2018). Direct economic compensation includes basic salary, performance, allowance, incentive salary, etc. (Bai and Luo, 2016), while indirect economic compensation refers to welfare reflected in endowment insurance, housing, transportation subsidies and clothing. The overall compensation structure includes not only economic compensation in the form of money but also non-economic compensation (Xing et al., 2017). Non-economic remuneration can be divided into direct and indirect non-economic compensation. Direct non-economic compensation is embodied in social status, interpersonal relationships, leadership, working environment, etc. Indirect non-economic compensation includes personal development and promotion opportunities, job achievements, and employee respect (Yang, 2010; Liu, 2011).

Currently, research on compensation incentives mainly focuses on the methods and effects. Compensation incentives mostly start with the comprehensive compensation structure, and studies show that fixed salaries, benefits (Dale-Olsen, 2006), allowances, career achievement, training conditions (Cui and Zou, 2013), job satisfaction, social status (Hulkko-Nyman et al., 2012), commercial insurance, and performance pay (Johnston, 2020) have emerged as effective ways to motivate employees. The compensation incentive effect can be measured based on employees’ feelings of motivation, effort, willingness to resign, feelings of motivation and career satisfaction (Jiang and Du, 2014). Therefore, the types of research objects must be considered in future research to select the most appropriate measurement of the compensation incentive effect.

## Theoretical hypotheses and index selection

### Theoretical hypotheses

Incentive theory indicates that managers should try their best to meet the diversified needs of the managed individuals and achieve salary incentives by improving salary design in salary management. As a special group, athletes are characterized by pragmaticism, patriotism, development, enjoyment, aggressiveness, and cooperation. These personality characteristics determine their diversified needs, such as the desire for good salaries and benefits, the need to satisfy the professional demand for national glory and independent training, and the hope to obtain good development opportunities and harmonious interpersonal relationships. The diversity of demand characteristics determines the comprehensiveness of the pay structure.

Therefore, this study further clarifies that the compensation defined in the evaluation of athletes’ compensation incentive effect is comprehensive compensation, including direct economic compensation, indirect economic compensation, direct non-economic compensation, and indirect non-economic compensation. Specifically, direct economic compensation includes basic remuneration, bonus income, and subsidies; indirect economic compensation consists of retirement placement, health insurance, and other welfare policies; direct non-economic compensation includes social status, leadership attention, favorable training environment and sound interpersonal relationships; indirect non-economic compensation includes athletes’ career fulfillment, training autonomy and sufficient opportunities for development and promotion. It is difficult to accurately measure the performance generated by athletes in the study of the compensation incentive effect for athletes. Thus, the compensation incentive effect of athletes is analyzed based on incentive theory, including pay incentive feelings and effects. According to the relevant research results of behavioral science, the compensation incentive effect is positively correlated with compensation satisfaction (Yang et al., 2017) and willingness to work hard (Ji and Cui, 2021). It is believed that the greater the compensation satisfaction of athletes, the stronger their willingness to train hard and the higher the compensation incentive effect.

The theoretical hypotheses for the compensation incentive effects of athletes are proposed, as shown in Table 1.

**Table 1:** Theoretical hypotheses of athletes’ compensation incentive effect.

Number	Hypothetical content
**H1**	Athletes’ direct economic compensation satisfaction exerts a significant positive impact on compensation incentive effects.
**H2**	Athletes’ indirect economic compensation satisfaction exerts a significant positive impact on compensation incentive effects.
**H3**	Athletes’ direct non-economic compensation satisfaction exerts a significant positive impact on compensation incentive effects.
**H4**	Athletes’ indirect non-economic compensation satisfaction exerts a significant positive impact on compensation incentive effects.

### Selection of evaluation indexes

According to the theoretical hypotheses, the compensation incentive effect of athletes (η) is affected by athlete’s direct economic compensation satisfaction (ζ_1_), indirect economic compensation satisfaction (ζ_2_), direct non-economic compensation satisfaction (ζ_3_), and indirect non-economic compensation satisfaction (ζ_4_). Therefore, according to the needs of athletes, 12 indicators covering four forms of compensation (X_1_–X_12_) were selected as the specific evaluation indicators of athletes’ compensation incentive effect. Additionally, we added two measurement indicators (Y_1_ and Y_2_) of the compensation incentive effect. An evaluation index system of athletes’ compensation incentive effect was established, with five latent variables and 14 measurement indicators. Detailed items can be found in the Appendix A.

Based on the evaluation indexes of athletes’ compensation incentive effect, data of each variable were tested for Kaiser–Meyer–Olkin (KMO) and Bartlett’s sphericity, where KMO = 0.786, and the approximate chi-square value was large with *p* < 0.001 (Table 2). This result indicated a significant correlation among the evaluation indicators of athletes’ compensation incentive effect, which is suitable for factor analysis. When extracting the common factor for the measurement indicators of athletes’ compensation incentive effect, it was found that the common degree of the interpersonal relationship (X9) was 0.344 < 0.7. Thus, the variable was deleted. Finally, an evaluation index system of athletes’ compensation incentive effect with five latent variables and 13 measurement indicators was formed.

**Table 2:** Test results of Kaiser–Meyer–Olkin and Bartlett on theoretical indicators for the evaluation of athletes’ compensation incentive effect.

KMO value	Bartlett’s sphericity test		
	Approximate chi-square value	df	*p*
0.786	2088.47	91	<0.001

## Research process and methods

### Questionnaire design

The questionnaire was designed based on scientific, systematic, easily accessible and non-oriented principles and was combined with changes in the salary structure of athletes in China. The questionnaire consisted of two parts: (a) basic personal information; (b) athletes’ perceptions and satisfaction with their salaries. In the first part, basic personal information included name, gender, age, training years, level, program, monthly income and region. According to relevant sources (Wen, 2006; Chen, 2010; Lazear, 2018), the second part contained 14 items of satisfaction measurement. A 5-point Likert scale was used to measure athletes’ salary satisfaction, with “1” (strongly dissatisfied), “2” (somewhat dissatisfied), “3” (neither satisfied nor dissatisfied), “4” (somewhat satisfied), and “5” (strongly satisfied).

### Data collection and processing

#### Data collection

To ensure the recovery rate and efficiency of questionnaires, they were distributed and collected at the Chenggong Training Base and Ridge Training Base in Kunming, Yunnan, China; Ersha Sports Training Center and CBA Training Venue in Guangzhou, Guangdong, China. A total of 400 questionnaires were distributed, and 390 were collected. Among them, 38 invalid questionnaires were excluded, and 352 valid questionnaires were eventually collected, with a recovery rate of over 90%. Due to the regional differences in China’s economic development level, athletes from Guangdong, Liaoning, Hunan, Jilin, Gansu and Yunnan provinces, representing the eastern, central and western regions, were selected as the subjects of the survey. The sports investigated included athletics, fencing, cycling, swimming, gymnastics, badminton, table tennis, basketball and soccer.

#### Research methods

The association between the analyzed variables cannot be verified simply by traditional regression analysis methods because they cannot be measured directly. Therefore, in this study, SPSS26.0 software and AMOS24.0 software were used to test and evaluate the validity of the scale and the overall model by structural equation modeling (Youssef et al., 2017; Wang et al., 2022).

## Results

### Demographic statistics

The information distribution of the samples is shown in Appendix B. Male athletes (60.5%) are more than female athletes (39.5%). Their age is mainly concentrated between 19 and 24 years old, accounting for 66.7% of the total. The years of training are more evenly distributed. From the perspective of level, national-level athletes and master sportsmen account for 45.7 and 44.9%, respectively. In terms of projects, athletics is regarded as the basic sport, with the highest proportion of 45.7%. Additionally, 66.7% of samples have a monthly income of 2001–4,000 yuan. In terms of region, athletes from the eastern region occupy the highest percentage (72.4%). The distribution of sample objects is reasonable.

### Model construction

#### Model specification

**Figure 1:**
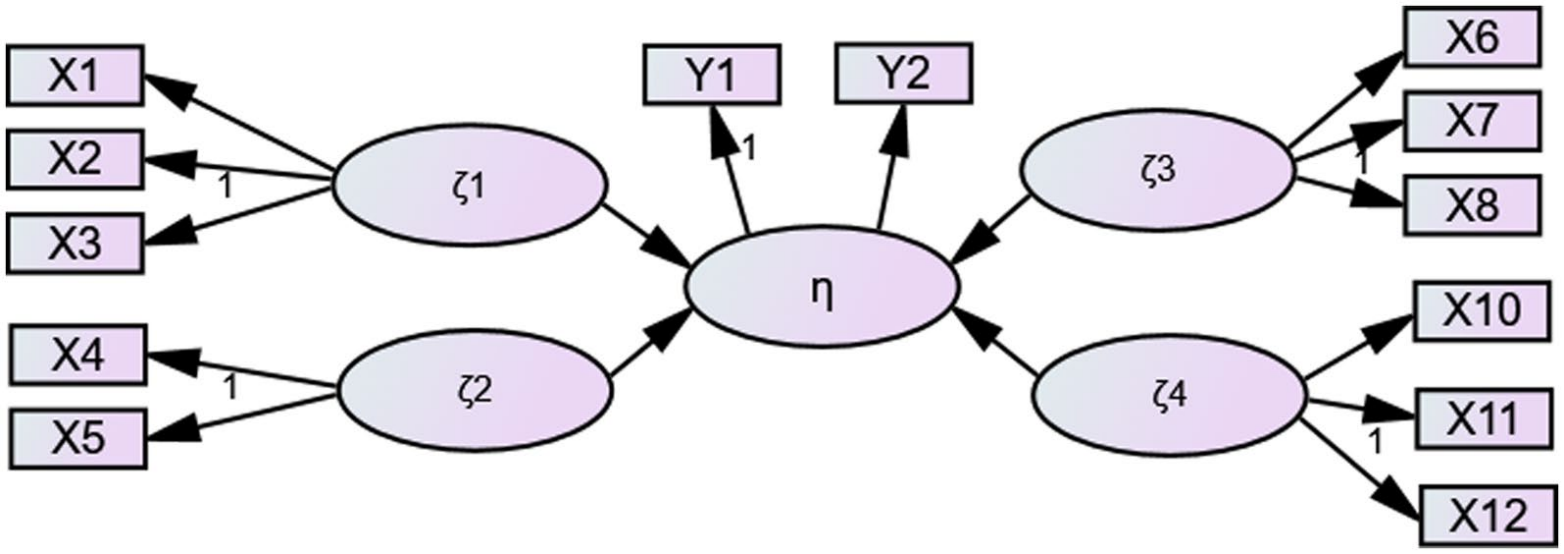
The model of athletes’ compensation incentive effect.

Based on research hypotheses, a structural equation model of factors that determines athletes’ compensation incentive effect was developed. The model possesses 5 latent variables and 13 observed items, as indicated in Figure 1. The athletes’ compensation incentive effect (η) is assumed to be determined by four dimensions of the compensation structure: direct economic compensation satisfaction (ζ_1_), indirect economic compensation satisfaction (ζ_2_), direct non-economic compensation satisfaction (ζ_3_), and indirect non-economic compensation satisfaction(ζ_4_). In addition, η, ζ_1_, ζ_2_, ζ_3_, and ζ_4_ are latent variables that cannot be directly measured. X_1_-X_12_ (except X_9_) and Y_1_-Y_2_ are the observed variables that can be directly measured, i.e., items in the questionnaire.

#### Model identification

In this paper, the t-rule is used for identifying the equation model. In the initial model, *p* = 11, *q* = 12, and the number of distinct parameters to be estimated are 36. Then, $\frac{1}{2}\left(p+q\right)\left(p+q+1\right)=91$ , $df=\frac{1}{2}\left(p+q\right)\left(p+q+1\right)-t=55>0$ , indicating that the model can be identified and is over-identified.

#### Model fitness test

##### Reliability and validity analysis

In the factor analysis approach in SEM, we first used confirmatory factor analysis to examine the construct validity. The scale of all variables was included in the confirmatory factor analysis. The method uses Cronbach’s Alpha coefficient and composite reliability (CR) to determine the reliability of the questionnaire and the internal consistency between variables (Shi et al., 2022; Wang et al., 2022). If the alpha value is greater than 0.7, a combined reliability of more than 0.6 indicates internal consistency between the observed and latent variables. Validation shows that both Cronbach’s alpha and CR values exceed 0.7 (Table 3), which means its internal consistency is suitable. Then, when evaluating the validity of the scales, we used the factor loading (FL) and the average variance extracted (AVE) of each variable, where FL was greater than 0.6, and the AVE was >0.5 (Hair et al., 2011; Wen et al., 2019; Luque-Reca et al., 2022). The validation data showed that the FL and AVE of each variable were higher than the standard values (Table 3), indicating that the questionnaire had good convergent validity and passed the validity test.

**Table 3:** Results of confirmatory factor analysis.

Variable	FL	Cronbach’s α	Ave	CR
Direct economic compensation satisfaction (ζ_1_)		0.768	0.568	0.795
Basic salary (X_1_)	0.600			
Bonus income (X_2_)	0.825			
Subsidy assistance (X_3_)	0.814			
Indirect economic compensation satisfaction (ζ_2_)		0.735	0.631	0.767
Retirement placement (X_4_)	0.937			
Medical insurance (X_5_)	0.620			
Direct non-economic compensation satisfaction (ζ_3_)		0.836	0.631	0.837
Social status (X_6_)	0.828			
Leadership attention (X_7_)	0.788			
Training condition (X_8_)	0.765			
Indirect non-economic compensation satisfaction (ζ_4_)		0.880	0.717	0.884
Career achievement (X_10_)	0.825			
Training autonomy (X_11_)	0.836			
Development and promotion opportunity (X_12_)	0.879			
Compensation incentive effect (η)		0.834	0.720	0.837
Salary incentive feeling (Y_1_)	0.797			
Effort will (Y_2_)	0.897			

FL, factor loading; CR, composite reliability; Ave, average variance extracted.

##### Overall model fit analysis

On the basis of testing the reliability and validity of the measurement model, we used the model fit index to judge the consistency between our hypothesis model and survey data. Generally, the ratio of the chi-square to the degrees of freedom (χ^2^/df), GFI (goodness of fit index), AGFI (adjusted goodness of fit index), RMSEA (root mean square residual), NFI (normed fit index), CFI (comparative fit index), etc. were considered (Zito et al., 2019). The closer the GFI and AGFI are to 1, the better the model fit is, and their acceptance criteria are above 0.9 (Li et al., 2021). Other acceptance criteria for the goodness-of-fit are detailed in Table 4. The results showed that the fit coefficients of the model indicators all exceeded the acceptance criteria, indicating that the overall model fit was good, i.e., the theoretical model and the data obtained in this study could be adapted.

**Table 4:** Fitting coefficients of the model indicators.

	(χ^2^/df)	GFI	AGFI	RMSEA	NFI	CFI	IFI
Judgment standard	1–3	>0.9	>0.9	<0.08	>0.9	>0.9	>0.9
Measured value	2.14	0.953	0.922	0.057	0.942	0.968	0.968
Acceptance level	Good	Very good	Very good	Good	Very good	Very good	Very good

#### Validation of model hypotheses

**Figure 2:**
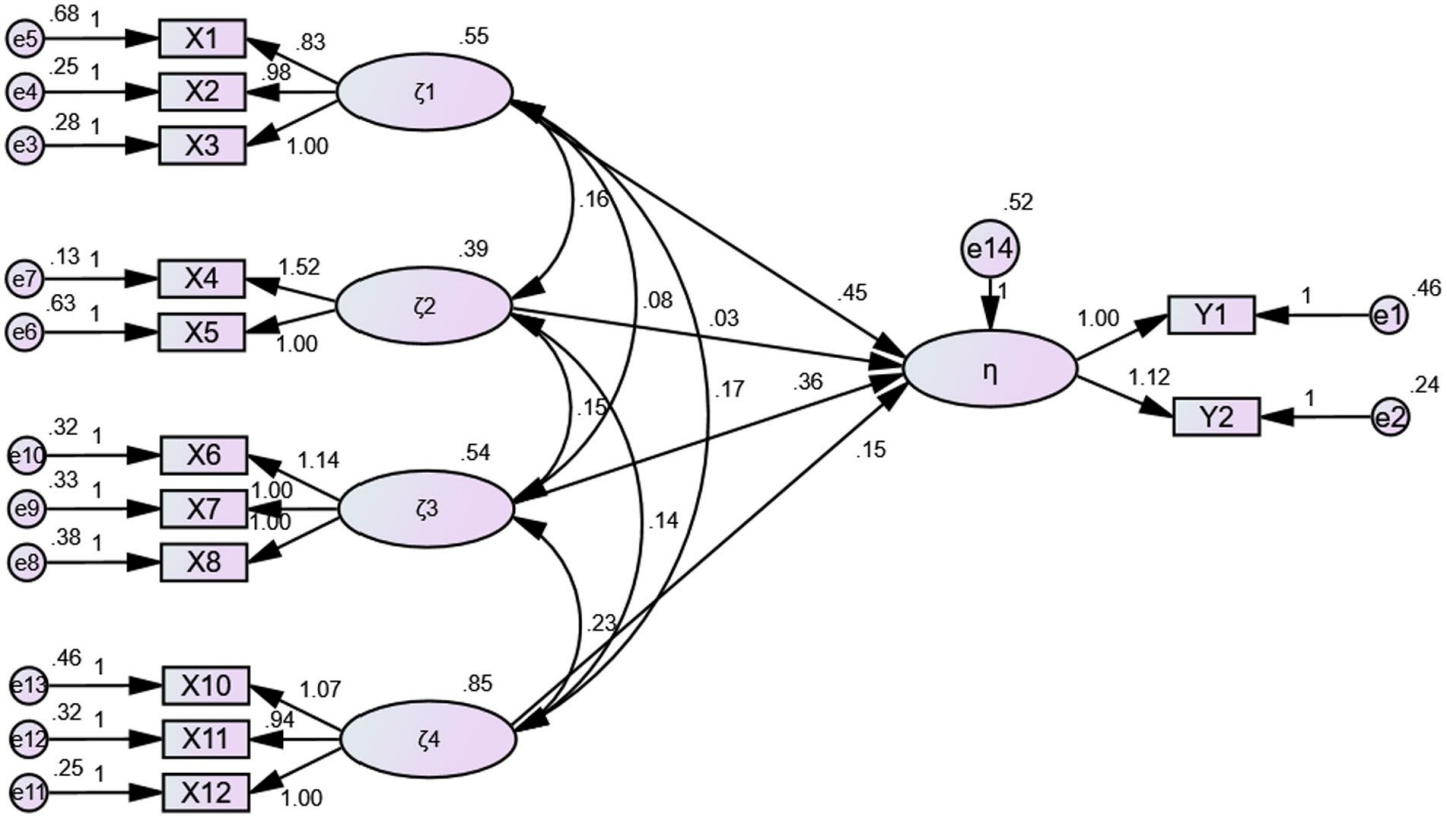
The SEM of athletes’ compensation incentive effect.

As shown in Table 5 and Figure 2, direct economic compensation and direct non-economic compensation show the greatest influence on compensation incentives (confidence level, 0.001). Indirect non-economic compensation positively impacts compensation incentives (confidence level, 0.01). Thus, the research hypotheses H1, H3, and H4 are supported. However, the impact of indirect economic compensation on compensation incentives has not reached a statistically significant level. Therefore, hypothesis “H2” is not supported.

**Table 5:** Model path coefficient of structural equation model and hypothesis test results.

The path	Nonstandardized coefficient	Standardized coefficient	S.E.	C.R.	P	Results
H1: η < −ζ_1_	0.447	0.371	0.081	5.517	***	support
H2: η < −ζ_2_	0.027	0.019	0.088	0.311	0.756	nonsupport
H3: η < −ζ_3_	0.361	0.297	0.080	4.519	***	support
H4: η < −ζ_4_	0.146	0.150	0.058	2.499	**	support
Y_1_ < −η	1.000	0.797				
Y_2_ < −η	1.118	0.897	0.097	11.502	***	
X_3_ < −ζ_1_	1.000	0.814				
X_2_ < −ζ_1_	0.983	0.825	0.076	12.926	***	
X_1_ < −ζ_1_	0.828	0.600	0.079	10.464	***	
X_5_ < −ζ_2_	1.000	0.620				
X_4_ < −ζ_2_	1.515	0.937	0.253	5.978	***	
X_8_ < −ζ_3_	1.000	0.765				
X_7_ < −ζ_3_	0.998	0.788	0.072	13.774	***	
X_6_ < −ζ_3_	1.142	0.828	0.081	14.113	***	
X_12_ < −ζ_4_	1.000	0.879				
X_11_ < −ζ_4_	0.939	0.836	0.051	18.448	***	
X_10_ < −ζ_4_	1.070	0.825	0.059	18.180	***	

**Significance level < 0.01 (two-tailed); ***Significance level < 0.001.

### Determination of the weight coefficient

The structural equation model mentioned above clearly reflects the path coefficients between the latent and observed variables. The path coefficients are normalized to calculate the weight coefficients of the relevant variables. The weight coefficient determines the contribution of the influence factor. Therefore, analyzing weight coefficients can determine the main reasons affecting the level of compensation incentive effect and provide a realistic basis for scientifically formulating countermeasures to improve the compensation system for athletes. Based on the analysis, the path coefficients that have passed the hypothesis validation variables in the evaluation model—three latent variables (ζ_1,_ ζ_3,_ and ζ_4_) and nine observation variables (X_1,_ X_2,_ X_3,_ X_6,_ X_7,_ X_8,_ X_10_, X_11,_ and X_12_)—are normalized (Table 6). The normalization method is to add the standardized path coefficients of each dimension above and then divide the standardized path coefficients of each dimension by the sum of the dimension coefficients to obtain the corresponding weight coefficients (Zhang, 2015).

**Table 6:** Weight coefficients of each index of athletes’ compensation incentive effect.

Primary indicators	Secondary indicators		Three indicators
	Variables	Weight	Indicators	Weight
η	ζ_1_	0.454	Basic salary (X_1_)	0.268
			Bonus income (X_2_)	0.369
			Subsidy assistance (X_3_)	0.364
	ζ_3_	0.362	Social status (X_6_)	0.348
			Leadership attention (X_7_)	0.331
			Training condition (X_8_)	0.321
	ζ_4_	0.184	Career achievement (X_10_)	0.325
			Training autonomy (X_11_)	0.329
			Development and promotion opportunity (X_12_)	0.346

## Conclusion and discussion

The primary objective of the study is to determine the factors of the compensation incentive effect on athletes and to evaluate the compensation incentive effect produced by the current athlete pay system in the management implementation process. However, the impact of indirect economic compensation on compensation incentives has not reached a statistically significant level. Given that the hypothesis that indirect economic compensation satisfaction has a significant effect on the pay incentive effect was not verified, the athlete compensation incentive effect was evaluated in terms of direct economic compensation, direct non-economic compensation, and indirect non-economic compensation.

In this study, direct economic compensation satisfaction was found to be the most important factor of the compensation incentive effect on athletes, with the value of its standardized regression weight being 0.454 (*p* < 0.01). This result is consistent with the findings of the Goksu and Oz (2010) and Hauret and Williams (2019). Therefore, to enhance athletes’ compensation incentive effect, managers should focus on ζ_1_, i.e., the improvement of direct economic compensation satisfaction. For the weight coefficients of the three obvious variables that affect the satisfaction of athletes’ direct economic compensation, the effect of bonus income and subsidy assistance on direct economic salary satisfaction are relatively close. Both are higher than the influence of basic salary. Since the basic salary of athletes is only the basic guarantee of their lives, their main economic income actually comes from subsidies from the state and government and bonus income from various competitions. Therefore, managers should conduct sufficient market research when formulating the basic salary standard of athletes, link their basic salary to the local minimum wage line, and strengthen the allocation of diversified bonuses and allowances on the basis of ensuring their basic living needs. Managers should avoid a simple “one-size-fits-all” approach to supply but formulate detailed and reasonable bonuses for athletes, impartially and comprehensively considering various factors such as athlete’s age, length of service, level, athletic performance and effort.

In this study, direct non-economic compensation satisfaction was found to be the second most important factor of compensation incentive effect on athletes with a weight coefficient of 0.355, second only to the influence of ζ_1_. Therefore, managers who improve the direct economic compensation structure of athletes also need to pay extra attention to the optimization of direct non-economic pay mechanisms to improve athletes’ compensation satisfaction. However, it is worth noting that ζ_3_ is not a single-dimensional concept but a multidimensional construct determined by many factors such as social status, leadership importance, and training conditions. Social status, leadership importance and training condition have a great impact on the weights of the three variables reflecting athletes’ direct non-financial compensation satisfaction (ζ_3_). Therefore, managers should pay full attention to them and improve the social status of athletes through the effect of public opinion to enhance their sense of collective honor in the practice of salary management. In addition, they should fully respect the will of individual athletes, adopt their opinions and suggestions, and strive to create a good external environment for their hard work and training by building a sound training environment.

The results of the weight coefficient show that indirect non-economic compensation satisfaction (ζ_4_) is the third important structure. It also has a significant effect on the compensation incentive effect of athletes. This result supports the study of the Hilliard and Boulton (2012), McConnell (2017), and Huang et al. (2022). Theoretically, the influence of ζ_4_ on η is minimal. However, in practice, it is found that the effect of ζ_4_ cannot be underestimated, especially for athletes with a strong sense of autonomy and independence and a sense of collective honor for the country. For athletes with a strong sense of autonomy and independence and a sense of collective honor for their country, the effect of ζ_4_ is sometimes even stronger than economic compensation. Therefore, in designing and optimizing the compensation structure of athletes, managers should not neglect to improve the satisfaction of indirect non-economic compensation (ζ_4_). The ζ_4_ is greatly impacted by the weight coefficients of three significant variables reflecting athletes’ satisfaction with indirect non-financial compensation, i.e., satisfaction with career achievement, satisfaction with training autonomy and satisfaction with promotion criteria. Therefore, government departments should strengthen public opinion and propaganda to enhance athletes’ professional achievement and autonomy to train actively for their country. Additionally, they should refine the athlete promotion evaluation system and comprehensively evaluate the athletes from multiple aspects, such as their cultural level and ideological and moral quality evaluation, to reshape the promotion evaluation system and promote the all-round development of the athletes’ quality, thus enhancing the athletes’ compensation incentive effect.

## Limitations and future research

This paper has two limitations. On the one hand, the data samples and sources are limited. This paper contains many details specific to the situation in China and the effects of the remuneration system on incentivizing athletes. Hence the recommendations are somewhat situational. Although the sample included athletes from different provinces and regions of China to increase the generalizability of the study, the number of samples can be increased and extended beyond athletes of the same country. On the other hand, athletes’ compensation incentive effect is influenced by multidimensional factors. When selecting latent and measurement variables, it is inevitable that some variables are difficult to quantify and are not included in the model, such as the fairness factor. Therefore, future research should focus on investigating the inclusion of more variables into the model and then improve the structural equation model.

## Data availability statement

The raw data supporting the conclusions of this article will be made available by the authors, without undue reservation.

## Author contributions

HZ: writing-original draft. HZ and ZL: design and/or conceptualization of the study. HZ, SZ, and FX: analysis and/or interpretation of the data. ML, RL, CX, and LZ: drafting and/or revising the manuscript. All authors contributed to the article and approved the submitted version.

## Conflict of interest

The authors declare that the research was conducted in the absence of any commercial or financial relationships that could be construed as a potential conflict of interest.

## Publisher’s note

All claims expressed in this article are solely those of the authors and do not necessarily represent those of their affiliated organizations, or those of the publisher, the editors and the reviewers. Any product that may be evaluated in this article, or claim that may be made by its manufacturer, is not guaranteed or endorsed by the publisher.
