# Supplementary material for: The compensation incentive effect of athletes: A structural equation model
Source: Front Psychol. 2022 Nov 18;13:1034855. doi: 10.3389/fpsyg.2022.1034855 (PMC9716138; doi:10.3389/fpsyg.2022.1034855)
Supplement: Supplementary file 1 [file Data_Sheet_1.doc]

**Table1 Theoretical hypotheses of athletes’ compensation incentive effect**

| **Number** | **Hypothetical content** |
| --- | --- |
| H1 | Athletes’ direct economic compensation satisfaction exerts a significant positive impact on compensation incentive effects. |
| H2 | Athletes’ indirect economic compensation satisfaction exerts a significant positive impact on compensation incentive effects. |
| H3 | Athletes’ direct non-economic compensation satisfaction exerts a significant positive impact on compensation incentive effects. |
| H4 | Athletes’ indirect non-economic compensation satisfaction exerts a significant positive impact on compensation incentive effects. |

**Table2 Test results of Kaiser–Meyer–Olkin and Bartlett on theoretical indicators for the evaluation of athletes’ compensation incentive effect**

| KMO value | Bartlett’s sphericity test | | |
| --- | --- | --- | --- |
|  | Approximate chi-square value | df | *p* |
| 0.786 | 2088.47 | 91 | <0.001 |

**Table 3 Results of Confirmatory Factor Analysis**

| **Variable** | **FL** | **Cronbach’s α** | **Ave** | **CR** |
| --- | --- | --- | --- | --- |
| Direct economic compensation satisfaction (ζ_1_) |  | 0.768 | 0.568 | 0.795 |
| Basic salary (X_1_) | 0.600 |  |  |  |
| Bonus income (X_2_) | 0.825 |  |  |  |
| Subsidy assistance (X_3_) | 0.814 |  |  |  |
| Indirect economic compensation satisfaction (ζ_2_) |  | 0.735 | 0.631 | 0.767 |
| Retirement placement (X_4_) | 0.937 |  |  |  |
| Medical insurance (X_5_) | 0.620 |  |  |  |
| Direct non-economic compensation satisfaction (ζ_3_) |  | 0.836 | 0.631 | 0.837 |
| Social status (X_6_) | 0.828 |  |  |  |
| Leadership attention (X_7_) | 0.788 |  |  |  |
| Training condition (X_8_) | 0.765 |  |  |  |
| Indirect non-economic compensation satisfaction (ζ_4_) |  | 0.880 | 0.717 | 0.884 |
| Career achievement (X_10_) | 0.825 |  |  |  |
| Training autonomy (X_11_) | 0.836 |  |  |  |
| Development and promotion opportunity (X_12_) | 0.879 |  |  |  |
| Compensation incentive effect (η) |  | 0.834 | 0.720 | 0.837 |
| Salary incentive feeling (Y_1_) | 0.797 |  |  |  |
| Effort will (Y_2_) | 0.897 |  |  |  |

Note:FL:factor loading; CR: Composite Reliability; Ave: Average Variance Extracted

Note:FL:factor loading; CR: Composite Reliability; Ave: Average Variance Extracted;

**Table 4 Fitting coefficients of the model indicators**

|  | **(χ^2^/df)** | **GFI** | **AGFI** | **RMSEA** | **NFI** | **CFI** | **IFI** |
| --- | --- | --- | --- | --- | --- | --- | --- |
| Judgment standard | 1-3 | >0.9 | >0.9 | <0.08 | >0.9 | >0.9 | >0.9 |
| Measured value | 2.14 | 0.953 | 0.922 | 0.057 | 0.942 | 0.968 | 0.968 |
| Acceptance level | Good | Very good | Very good | Good | Very good | Very good | Very good |

**Table5 Model path coefficient of structural equation model and hypothesis test results**

| **The path** | **Nonstandardized coefficient** | **Standardized coefficient** | **S.E.** | **C.R.** | **P** | **Results** |
| --- | --- | --- | --- | --- | --- | --- |
| H1: η<--ζ_1_ | 0.447 | 0.371 | 0.081 | 5.517 | *** | support |
| H2: η<--ζ_2_ | 0.027 | 0.019 | 0.088 | 0.311 | 0.756 | nonsupport |
| H3: η<--ζ_3_ | 0.361 | 0.297 | 0.080 | 4.519 | *** | support |
| H4: η<--ζ_4_ | 0.146 | 0.150 | 0.058 | 2.499 | ** | support |
| Y_1_<--η | 1.000 | 0.797 |  |  |  |  |
| Y_2_<--η | 1.118 | 0.897 | 0.097 | 11.502 | *** |  |
| X_3_<--ζ_1_ | 1.000 | 0.814 |  |  |  |  |
| X_2_<--ζ_1_ | 0.983 | 0.825 | 0.076 | 12.926 | *** |  |
| X_1_<--ζ_1_ | 0.828 | 0.600 | 0.079 | 10.464 | *** |  |
| X_5_<--ζ_2_ | 1.000 | 0.620 |  |  |  |  |
| X_4_<--ζ_2_ | 1.515 | 0.937 | 0.253 | 5.978 | *** |  |
| X_8_<--ζ_3_ | 1.000 | 0.765 |  |  |  |  |
| X_7_<--ζ_3_ | 0.998 | 0.788 | 0.072 | 13.774 | *** |  |
| X_6_<--ζ_3_ | 1.142 | 0.828 | 0.081 | 14.113 | *** |  |
| X_12_<--ζ_4_ | 1.000 | 0.879 |  |  |  |  |
| X_11_<--ζ_4_ | 0.939 | 0.836 | 0.051 | 18.448 | *** |  |
| X_10_<--ζ_4_ | 1.070 | 0.825 | 0.059 | 18.180 | *** |  |

*Significance level <0.05; **Significance level <0.01 (two-tailed); ***Significance level <0.001.

**Table 6 Weight coefficients of each index of athletes’ compensation incentive effect**

| **Primary indicators** | **Secondary indicators** | | **Three indicators** |  |
| --- | --- | --- | --- | --- |
|  | **Variables** | **Weight** | **Indicators** | **Weight** |
| η | ζ_1_ | 0.454 | Basic salary (X_1_) | 0.268 |
|  |  |  | Bonus income (X_2_) | 0.369 |
|  |  |  | Subsidy assistance (X_3_) | 0.364 |
|  | ζ_3_ | 0.362 | Social status (X_6_) | 0.348 |
|  |  |  | Leadership attention (X_7_) | 0.331 |
|  |  |  | Training condition (X_8_) | 0.321 |
|  | ζ_4_ | 0.184 | Career achievement (X_10_) | 0.325 |
|  |  |  | Training autonomy (X_11_) | 0.329 |
|  |  |  | Development and promotion opportunity (X_12_) | 0.346 |

**APPENDIX A Evaluation indexes of athletes’ compensation incentive effect**

| **Latent variables** | **Measurement indicators** |
| --- | --- |
| Compensation incentive effect (η) | Salary incentive feeling (Y_1_): Do you think the current income level motivates athletes? |
|  | Effort will (Y_2_): Are you willing to train hard to achieve results for yourself and your team? |
| Direct economic compensation satisfaction (ζ_1_) | Basic salary (X_1_): Are you satisfied with your current basic salary? |
|  | Bonus income (X_2_): Are you satisfied with the current bonus income? |
|  | Subsidy assistance (X_3_): Are you satisfied with the current subsidy assistance? |
| Indirect economic compensation satisfaction (ζ_2_) | Retirement placement (X_4_): Are you satisfied with the current retirement placement policy? |
|  | Medical insurance (X_5_): Are you satisfied with the current medical insurance? |
| Direct non-economic compensation satisfaction (ζ_3_) | Social status (X_6_): Are you satisfied with the current recognition of athletes’ social status? |
|  | Leadership attention (X_7_): Are you satisfied with the importance your leaders place on you or the team? |
|  | Training condition (X_8_): Are you satisfied with the training environment and conditions?  Interpersonal relationship (X_9_): Would you like to describe a sports team as a “family” where people feel they belong? |
| Indirect non-economic compensation satisfaction (ζ_4_) | Career achievement (X_10_): How fulfilling is the project you are currently working on? |
|  | Training autonomy (X_11_): Do leaders or coaches value your training ideas? |
|  | Development and promotion opportunity (X_12_): Do you see a future for your sports team? |

**APPENDIX B Characteristics of the sample**

| **Basic information** | **Category** | **Frequency** | **%** |
| --- | --- | --- | --- |
| Gender | Male | 213 | 60.5 |
|  | Female | 139 | 39.5 |
| Age | <=18 | 53 | 15.1 |
|  | 19–24 | 235 | 66.7 |
|  | >=25 | 64 | 18.2 |
| Training years | <=2 | 93 | 26.4 |
|  | 2–4 | 120 | 34.1 |
|  | 4–6 | 81 | 23 |
|  | >=6 | 58 | 16.5 |
| Level | Master sportsman | 158 | 44.9 |
|  | National-level athletes | 161 | 45.7 |
|  | Second-level athletes | 33 | 9.4 |
| Program | Athletics | 161 | 45.7 |
|  | Fencing | 18 | 5.1 |
|  | Cycling | 27 | 7.7 |
|  | Swimming | 22 | 6.3 |
|  | Gymnastics | 26 | 7.4 |
|  | Badminton | 32 | 9.1 |
|  | Table tennis | 6 | 1.7 |
|  | Basketball | 18 | 5.1 |
|  | Soccer | 42 | 11.9 |
| Monthly income | <=2000 | 59 | 16.8 |
|  | 2001–4000 | 235 | 66.7 |
|  | 4001–6000 | 12 | 3.4 |
|  | >6000 | 46 | 13.1 |
| Region | Eastern region | 255 | 72.4 |
|  | Central region | 38 | 10.8 |
|  | Western region | 59 | 16.8 |
